# Adipose-Derived Mesenchymal Stem Cells Applied in Fibrin Glue Stimulate Peripheral Nerve Regeneration

**DOI:** 10.3389/fmed.2019.00068

**Published:** 2019-04-09

**Authors:** Ruslan Masgutov, Galina Masgutova, Adelya Mullakhmetova, Margarita Zhuravleva, Anna Shulman, Alexander Rogozhin, Valeriya Syromiatnikova, Dina Andreeva, Alina Zeinalova, Kamilla Idrisova, Cinzia Allegrucci, Andrey Kiyasov, Albert Rizvanov

**Affiliations:** ^1^OpenLab Gene and Cell Technologies, Institute of Fundamental Medicine and Biology, Kazan Federal University, Kazan, Russia; ^2^Department of Orthopaedics, Republic Clinical Hospital, Kazan, Russia; ^3^Scientific Department, Republic Clinical Hospital, Kazan, Russia; ^4^Department of Neurology, Kazan State Medical Academy, Branch of Russian Medical Academy of Postgraduate Education, Kazan, Russia; ^5^School of Veterinary Medicine and Science, University of Nottingham, Nottingham, United Kingdom

**Keywords:** rat, sciatic nerve injury, MSCs, ADSCs, fibrin glue

## Abstract

Mesenchymal stem cells (MSCs) hold a great promise for cell therapy. To date, they represent one of the best choices for the treatment of post-traumatic injuries of the peripheral nervous system. Although autologous can be easily transplanted in the injured area, clinical advances in this filed have been impaired by lack of preservation of graft cells into the injury area after transplantation. Indeed, cell viability is not retained after injection into the blood stream, and cells injected directly into the area of injury either are washed off or inhibit regeneration through scar formation and neuroma development. This study proposes a new way of MSCs delivery to the area of traumatic injury by using fibrin glue, which not only fixes cells at the site of application but also provides extracellular matrix support. Using a sciatic nerve injury model, MSC derived from adipose tissue embedded in fibrin glue were able to enter the nerve and migrate mainly retrogradely after transplantation. They also demonstrated a neuroprotective effect on DRG L5 sensory neurons and stimulated axon growth and myelination. Post-traumatic changes of the sensory neuron phenotype were also improved. Importantly, MSCs stimulated nerve angiogenesis and motor function recovery. Therefore, our data suggest that MSC therapy using fibrin glue is a safe and efficient method of cell transplantation in cases of sciatic nerve injury, and that this method of delivery of regeneration stimulants could be beneficial for the successful treatment of other central and peripheral nervous system conditions.

## Introduction

Peripheral neuropathy caused by trauma and prevalent disorders is a major medical problem worldwide. Due to anatomy of the peripheral nervous system, structure damage of peripheral nerve trunks is a rather common condition. Although nerve trunk injury is not a life-threatening condition, it is accompanied by a persistent complex of nociceptive, sensory, and motor as well as trophic changes that can result in long-lasting disability, with full recovery of lost extremity functions being difficult (1, 2). Therefore, new translational research is focusing on combined methods of tissue engineering and replacement cell therapy along with the advancements in microsurgical techniques to enable efficient restoration of damaged peripheral nerves in clinical practice (3–8).

When facilitating nerve structure recovery to reduce nerve tissue injury, the main focus is to use various organic glues that join the ends of the damaged nerve (9) or are part of nerve conduit (10). The use of fibrin glue is particularly promising as it has been shown to be non-invasive and not causing inflammation or granuloma formation in post-traumatic nerve reconstruction (11–13). Fibrin glue can also induce faster re-innervation and function recovery due to reduced amount of scar tissue and directed axon growth (14, 15).

After nerve injury, the release of pro-inflammatory cytokines causes fibroblast activation and collagen hyperplasia leading to scars, fibrosis, and neuroma formation. These pro-inflammatory and nerve growth factors are necessary for axon regeneration by maintaining the balance between collagen synthesis and degradation without inducing fibroblast activation (16). Therefore, targeted delivery of potential neurorestoration stimulants is extremely important and cell therapy can be used to accelerate axon growth and help sensory neurons to survive (17, 18). To this end, mesenchymal stem cells (MSCs) have been most widely used (19) as an autologous system able to stimulate nerve tissue regeneration by neurotrophic growth factor secretion (20) and guided differentiation to the neuronal and glial lineages (21, 22).

MSCs can replace damaged tissues, secrete biologically active compounds, including homing and signaling molecules as well as myelin components necessary for structural and functional recovery of nerve fibers (23). Importantly, they do not cause immune rejection after allogeneic transplantation (24). MSCs can be easily derived from various tissues and expanded in culture. A useful source is the adipose tissue as adipose derived stem cells (ADSCs) have demonstrated MSCs properties (19, 25), together with ease of availability, derivation, and safety (26).

In order to attain efficient regeneration, transplanted cells need to survive and function in the area of injury. Cell survival after their injection is influenced by many factors, including speed of cell injection, cell density and diameter of the needle (27, 28). Regardless of the method used to assess the survival rate, cell type, and transplantation model, it is widely accepted that only a small portion of transplanted cells survive after injection (29, 30). Indeed, cells can be lost in the systemic circulation, but also local cell delivery by injection can cause additional damage and lead to neuroma development (31). Therefore, it is necessary to address a number of key issues to improve local cell retention in the area of injury before cell therapy can become a viable treatment option for peripheral nerve injury. In this study, we used fibrin glue for cell delivery. This formulation demonstrates several advantages, being minimally invasive, helping to retain cells in the area of injury, providing cells with extracellular matrix support and additional physical connection of the nerve ends (glue effect). We show that transplantation of MSCs embedded in fibrin glue promotes regeneration through a neuroprotective effect on sensory neurons and stimulation of axon growth. We also demonstrate for the first time a positive effect on recovery of motor function after injury, thus paving the way for the use of fibrin glue and MSC cell therapy in the treatment of peripheral nerve damage.

## Materials and Methods

### MSC Derivation and Culture

MSC (ADSCs) were derived from the inguinal fold subcutaneous adipose tissue of healthy female Wistar rats (*n* = 5), as previously described (32). Subcutaneous fat tissue from different animals was obtained in equal volumes and immediately mixed for subsequent cultivation. Adipose tissue enzymatic disaggregation was achieved by incubation with type 1 collagenase (Biolot, Russia) at 37°C on a shaker for 1 h. Before and after the incubation the tissue was washed three times by centrifugation at 500 rpm for 5 min with DPBS solution (Dulbecco‘s Phosphate Buffered Saline, Paneco, Russia) containing 5% of penicillin and 5% of streptomycin. The cell pellet was resuspended and plated in culture in αMEM (Alpha Minimum Essential Medium, Invitrogen, USA) with 10% of FBS (Fetal Bovine Serum, Sigma, USA). Cultures were cultured and expanded in αMEM with 10% of FBS, 2 mM of L-glutamine (Sigma, USA) and penicillin and streptomycin (100 U/mL; 100 μg/mL) (Sigma, USA) using a MCO-15AC incubator (Sanyo, Japan) at 5% CO_2_ and 37°C.

Rat ADSCs immunophenotype was evaluated using flow cytometry. Cells were trypsinized using 0.25% trypsin (Sigma, USA) and incubated in phosphate-buffered saline for 45 min with conjugated antibodies anti-CD29 (BD, USA, 556049), anti-CD44 (BioLegend, USA, 103028), anti-CD90 (SantaCruz, USA, SC-53456), anti-CD34 (SantaCruz, USA, SC-51540), and anti-CD45 (SantaCruz, USA, SC-70686). Analysis was performed using a Guava Easy Cyte 8HT flow cytometer (Millipore, United States).

After confirmation of the MSCs immunophenotype, ADSCs were transduced using a recombinant lentivirus encoding eGFP (green fluorescent protein). Transdusction efficiency was assessed 48 h later on the basis of the number of eGFP positive cells using an Axio Observer Z1 fluorescence microscope (Carl Zeiss, Germany). eGFP-expressing cells were sorted using a FACS Aria III cell sorter (BD Biosciences, USA) and expanded in cultured until transplantation. A population of cells expressing ≥90% eGFP (ADSCs-eGFP) was used for cell transplantation.

### Experiments With Animals

Experiments were conducted using 62 white Wistar rats aged 4–6 months and weighting 200–300 g (GMBH “Nursery RAMTN,” Moscow, Russia). For study of regeneration processes on 30 and 60th days after injury experiment was carried out on 35 male rats. Twenty two male rats used for estimation of MSCs migration on 7 and 14th days after trauma. For obtaining of MSCs we used 5 female rats additionally. All animals were acclimatized for 2 weeks before starting the experiment. Animals were kept under standard vivarium conditions with the day/night mode 12/12, with free access to feed and water. Animals were kept and used for experimental procedures in accordance with the rules accepted by Kazan Federal University and approved by the Local Ethics Committee (Permit Number 5 of May 27, 2014). Animals were used in accordance to international bioethical standards defined by the International guiding principles for biomedical research involving animals (33), the EU directive 2010/63/EC and the 3Rs principles.

The autologous nerve graft (AG) was used as an experimental nerve model. The rats were deeply anesthetized with intraperitoneal injection of chloral hydrate solution (AppliChem, Germany) at a dose of 400 mg/kg in the water for injections. Surgical approach was made to the left sciatic nerve, then a 5 mm long diastasis was formed by transecting the nerve with two parallel incisions. The ends of the nerve were joined with an autologous nerve graft, strictly maintaining the nerve fiber growth cone, and sutured without tension with 4 Prolene 10.0 (Ethicon, USA) interrupted epineural sutures. The nerve was then covered with 200 μL of Tissucol-Kit fibrin glue (Baxter AG, Austria).

Stimulation of post-traumatic regeneration was achieved through intergender allotransplantation of ADSCs-eGFP. To this end, fibrin glue (FG) containing 1 million ADSCs-eGFP was applied to the nerve after suturing. After nerve manipulations the postoperative wound was closed in layers. Animals in 4 groups were compared: (1) experimental group (*n* = 5 on 7th, *n* = 5 on 14th, *n* = 5 on 30th, and *n* = 5 on 60th days after injury)—AG+FG+ADSCs—post-traumatic recovery by regeneration stimulation using ADSCs-eGFP; (2) active control group (*n* = 3 on 7th, *n* = 3 on 14th, *n* = 5 on 30th, and *n* = 5 on 60th days after injury)—AG+FG—post-traumatic recovery by nerve coverage with fibrin glue; (3) control group (*n* = 3 on 7th, *n* = 3 on 14th, *n* = 5 on 30th, and *n* = 5 on 60th days after injury)—AG—post-traumatic recovery under natural conditions with a autologous nerve graft. The animals in experimental groups were sacrified on 7th (*n* = 11), 14th (*n* = 11), 30th (*n* = 5), and on 60th (*n* = 5) days after injury; (4) intact group (*n* = 5)—intact animals with no damage to sciatic nerve.

Antibiotics and analgesics were administered as follows: 1 mL of gentamicin (25 mg/kg, Omela, Russian Federation) was injected intramuscularly for 7 consecutive days; buprenorphine (0.5 mg/kg) was injected subcutaneously for about 7 days after surgery to minimize pain.

### Assessment of Motor Function Recovery

To assess the grade of motor function recovery of the operated extremity, animals of all groups underwent a functional motor test to define the sciatic functional index (SFI) (34). The functional test was carried out once a week on day 7, 14, 21, 28, 35, 42, 49, and 56 after the injury. To this end, a run track with side walls was used (width−12 cm, length−45 cm, height−15 cm) allowing a rat to move only in one direction. Animals hind feet were covered in ink and footprints made on the run track covered with white paper were measured to calculate the SFI using the formula:

$$\begin{array}{l} \text{SFI}=[((\text{eTOF}-\text{nTOF})/\text{nTOF})+((\text{nPL}-\text{ePL})/\text{ePL})\\ +\;((\text{eTS}-\text{nTS})/\text{nTS})+((\text{eIT}-\text{nIT})/\text{nIT})]\times \text{55},\;\text{where} \end{array}$$

eTOF is the distance from the experimental foot toes to the intact foot toes of the subsequent footprint;

nTOF is the distance from the intact foot toes to the experimental foot toes of the subsequent footprint;

PL is the length of the footprint from the heel to the third toe of the same foot;

TS is the distance between the first and the fifth toes of the same foot;

IT is the distance between the second and the fourth toes of the same foot.

All the measurements were made both for the healthy foot footprint (*n*—normal) and the operated foot footprint (e—experimental; Supplementary Figure 1).

### Nerve Conduction Studies

Compound muscle action potentials (CMAPs) was used to assess sciatic nerve conduction of the rats both before and 30 and 60 days after the surgery. Electric muscle responses were registered using a MG-42 electromyograph (Hungary) combined with data computer analysis. After sedation, stimulant monopolar needle electrodes were inserted in the hip joint area and into the area of sciatic nerve projection. Stimulation was carried out with square-wave pulses lasting 1–2 ms. Stimulus intensity varied from 0.2 to 2 V. CMAP were registered by the monopolar needle electrodes inserted into the medial gastrocnemius: cathode was inserted into the center of the myogaster and the anode—into the tendon. Latency of the CMAP, response threshold, duration of the CMAP, and maximal amplitude were analyzed.

### Vascularization Assessment

Recovery of blood flow in the distal part of the sciatic nerve was assessed by the visualization of microcirculation perfusion 14 and 30 days after the surgery using a EasyLDI laser doppler (Aimago). To this end, small operating room repetitive approach to the sciatic nerve was carried out in the animals under intraperitoneal narcosis. The device's laser beam was pointed at the distal part of the nerve and changes in microcirculation parameters over time were analyzed using the function of perfusion unit assessment in real time. In the evaluation process, the laser beam sequentially scanned the tissue of the distal stump of the sciatic nerve, while circulating red blood cells generated Doppler components in scattered light, were picked up by a photodiode, and converted into an electrical signal proportional to tissue perfusion at each measurement point. The parameters were measured in absolute perfusion units (apu) according to manufacturer's instructions.

### Material Sampling and Morphological Assessment

Animals were sacrificed on day 7, 14, 30, and 60 after the surgery. Early periods were used to investigate the survival rate and migration ability of the transplanted cells. Samples collected on day 7 and 14 after the surgery were snap-frozen in liquid nitrogen with Neg50 Frozen Section Medium (Thermo Fisher Scientific, USA), and transferred and stored at −80°C. Sciatic nerve samples were cut into 6 mm thick longitudinal sections using a Cryo-Star HM560 freezing microtome (Thermo Fisher Scientific, USA). eGFP fluorescence was visualized using a LSM 780 laser scanning confocal microscope (Carl Zeiss, Germany). DAPI nuclear stain was used to assess the survival rate of transplanted cells. Hematoxylin and eosin staining used for estimation of morphological changes.

Late time points (day 30 and 60 after the injury the sciatic nerve) were used to investigate post-traumatic recovery by exposure of the L5 spinal ganglion on the operative side. Distal parts of sciatic nerves were used to assess the number of regenerating myelin fibers, whereas L5 spinal ganglia were used to assess the number of surviving neurons.

The exposed distal segments of the sciatic nerve were fixed in 2% glutaric dialdehyde, postfixed in 1% OsO_4_, embedded in epoxy resin (9 ml of Epon 812, 6 ml of DDSA, 4 ml of MNA, and 0.2 ml of DHP-30) and polymerized at 60°C. The samples were sliced in 2 μm thick semifine cross sections using a LKB 2088 ultramicrotome (Leitz, Germany) and sections stained with methylene blue. The number of myelin fibers was calculated by random sample analysis of 4 nerve segments (35) using a ICC50E light microscope (Leica, Germany) with 63 × 100 magnification and oil objective immersion using the ImageJ 1.48v program.

Totally exposed left L5 spinal ganglia after laminectomy were fixed in 4% buffered formaline and embedded in Histomix paraffin (Biovitrum, Russia) on a Tissue-Teck® TECTM 5 paraffin embedding station (Sakura Seiki, Japan). Paraffin blocks were cut into longitudinal 7 μm thick serial sections on a HM340E rotary microtome (Thermo Fisher Scientific, USA). Deparaffinized sections were stained to assess the post-traumatic reaction of spinal ganglion neurons. Every 5th serial section was stained with 5% azure and eosin according to Romanovsky (Minimed, Russia) to visualize neuronal nuclei for cell survival analysis. To assess the response of small diameter neurons, every 5th serial section of the spinal ganglion was stained with Isolectin-B4 fluorescence cytoplasmic marker [lectin from Bandeiraea simplicifolia (BSi-B4)] (IB-4) (Sigma, L2895, 1:200) and counterstained with the nuclear dye DAPI (Sigma) before visualization.

### Statistical Analysis

For all parameters of the given experimental groups, mean, and standard deviation were calculated and data presented as mean ± standard error of the mean (SEM). Student's *t*-test, one-way analysis of variance (ANOVA) with Tukey's test or two-way analysis of variance (ANOVA) were used for multiple comparisons between all experimental and control groups. The differences were considered significant if the probability threshold was < 0.05% (*p* < 0.05). Data were analyzed using the Origin 7.0 SR0 Software (OriginLab, Northampton, MA, USA).

## Results

**Figure 1:**
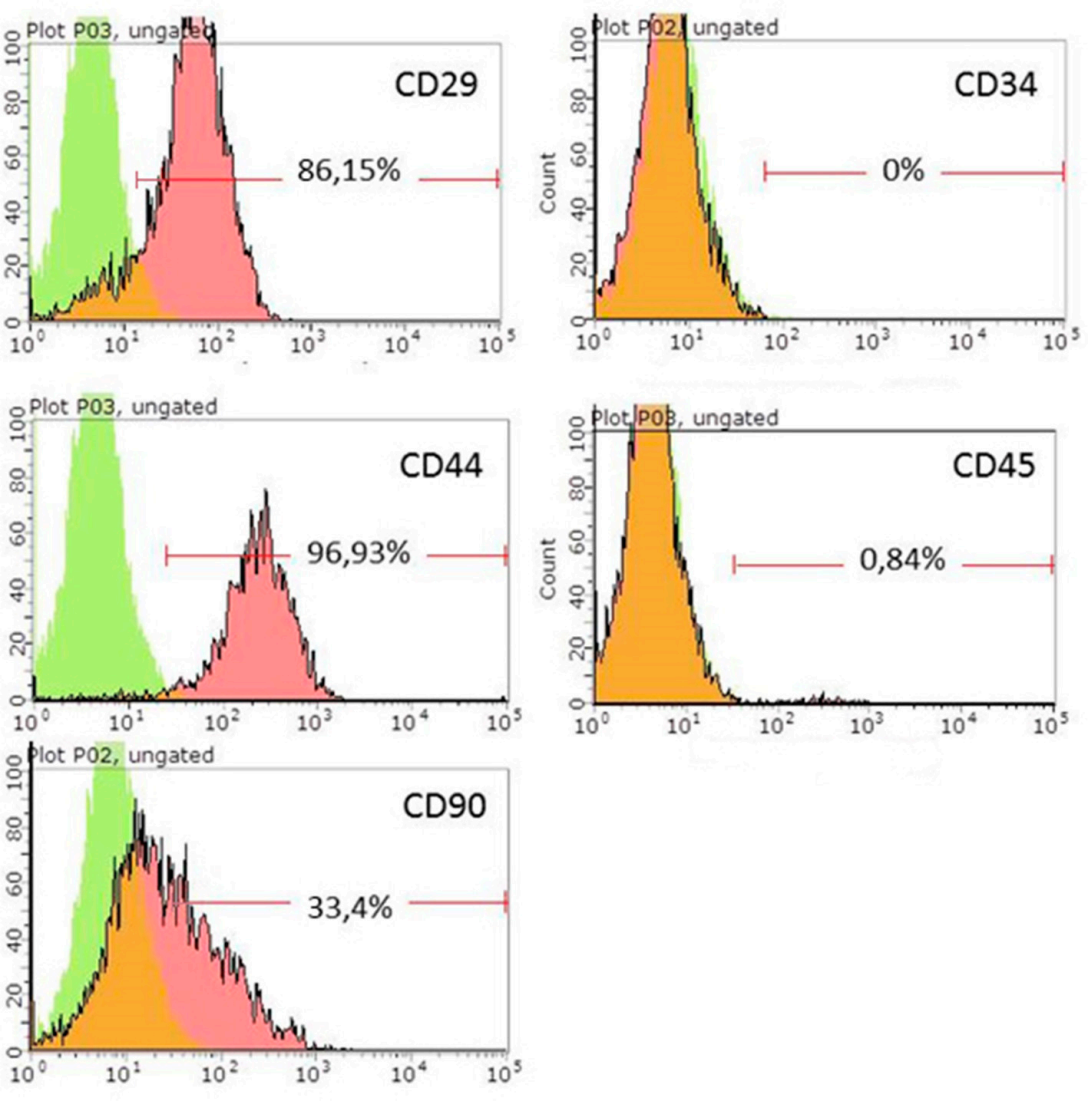
Flow cytometry analysis of rat ADSCs. Green, isotype control; Red, stained cells.

**Figure 2:**
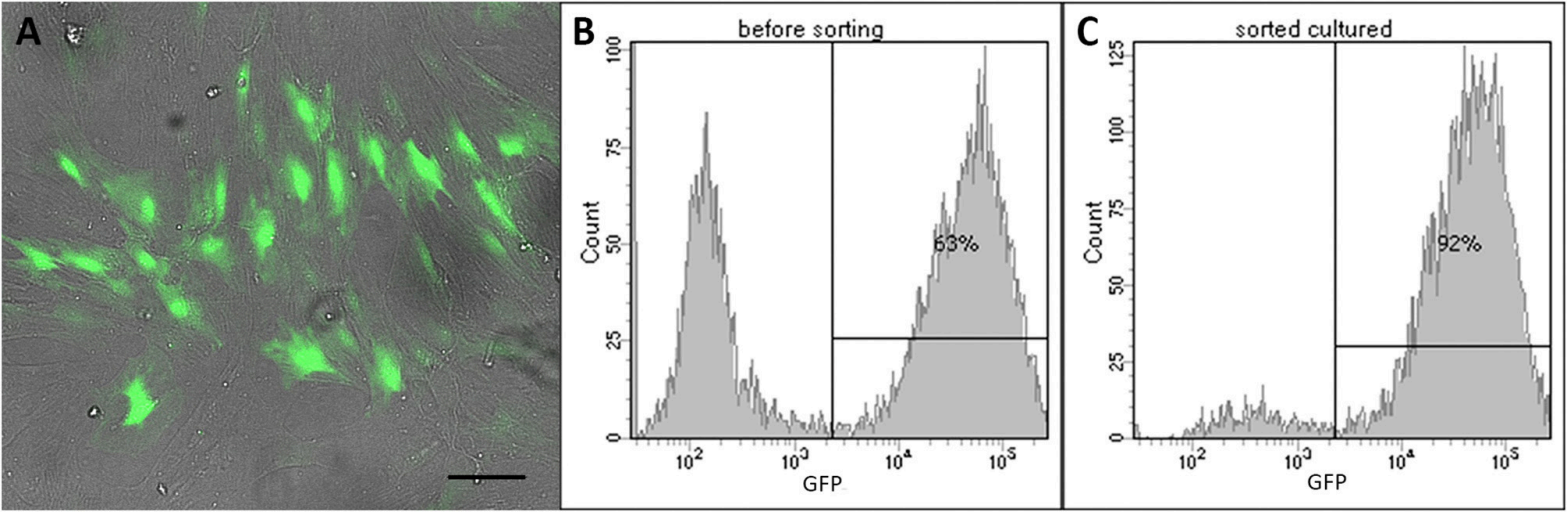
LV-eGFP transduced rat ADSCs. **(A)** e-GFP expression 48 h after transduction. Scale bar, 100 μm. **(B)** Flow cytometry analysis of e-GFP 48 h after transduction. **(C)** Flow cytometry analysis of e-GFP-sorted cells used for transplantation.

ADSCs isolated from rat adipose tissue had a fibroblast-like morphology and high proliferative activity. They expressed CD29, CD34, CD90 but not CD34, and CD45, confirming their MSC phenotype (Figure 1). Established ADSC cultures were then genetically modified to express e-GFP in order to generate a fluorescent line for cell tracing. Fluorescent green cells were detected 48 h after transduction and a population of ADSCs expressing ≥90% eGFP was obtained after sorting (Figure 2) Transplantation of fluorescent sorted cells allowed the visualization of MSC integration in the injured area.

**Figure 3:**
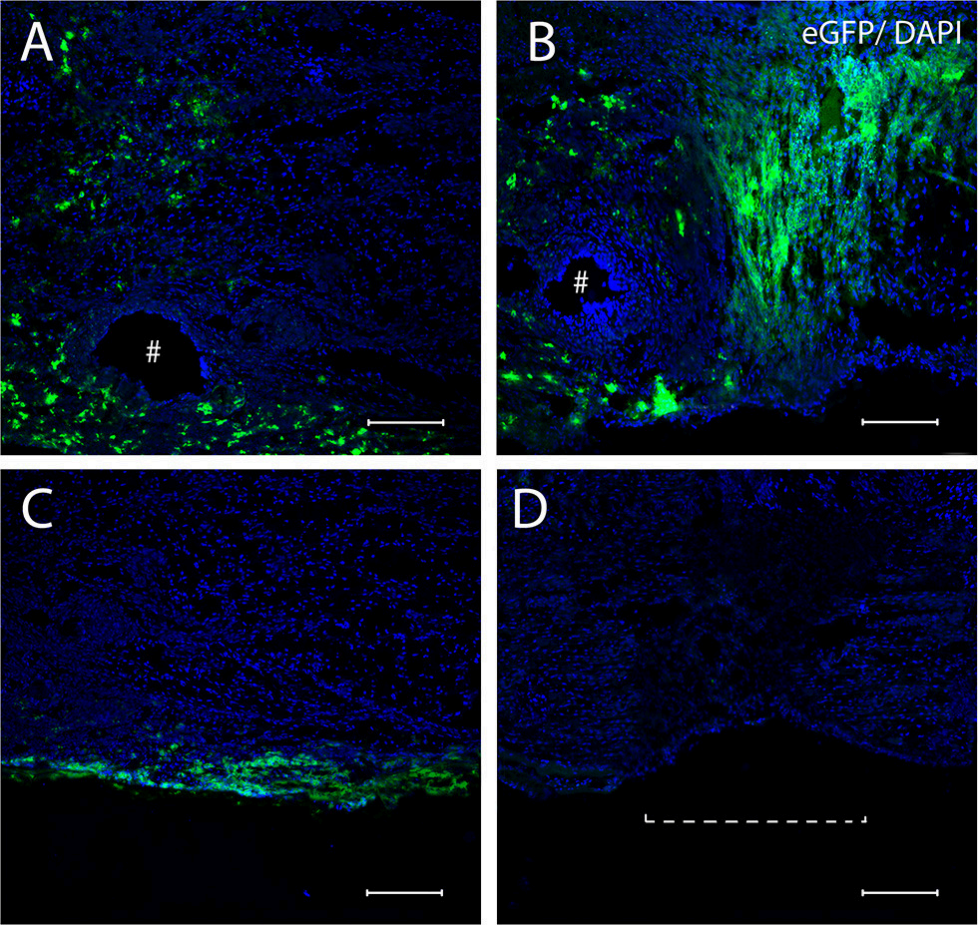
Confocal miscopy images of longitudinal sections of the rat sciatic nerve after 7 **(A,C)** and 14 **(B)** days in the AG+FG+ADSCs group and 14 days in the AG group **(D)**. **(A,B)** The area of anastomosis of sciatic nerve after 7 days **(A)** and 14 days **(B)** the penetration of ADSCs-eGFP through the suturing area is shown. **(C)** The inability of ADSCs-eGFP invasion through the intact epineurium is shown. **(D)** Absence of fluorescence in the AG-group is shown. #, the needle hole; a dotted line, nerve anastomosis. The green fluorescence indicates transplanted cells expressing e-GFP; blue fluorescence indicates cell nuclei stained with DAPI. Scale bars: 100 μm.

Examination of the sciatic nerve longitudinal sections after transplantation demonstrated ADSCs-eGFP on the surface of epineurium as part of the FG and in the sciatic nerve at day 7 and 14 of the autologous nerve repair. Although it was not possible to count the absolute number of cells under the epineurium, the e-GFP fluorescence enabled the assessment of the migration ability of ADSCs. Cells applied in fibrin glue on the surface sciatic nerve actively migrated under the epineurium through nerve sutures and moved mainly retrogradely (Figures 3A,B). After 7 days, cells migrated on average 1–2 mm and cells covered 5–7 mm distance 14 days after the application. However, ADSCs were not able to move through the intact epineurium connective tissue (Figure 3C). No fluorescence was found in the AG+FG group where the autologous nerve graft was covered with FG without ADSCs (Figure 3D).

**Figure 4:**
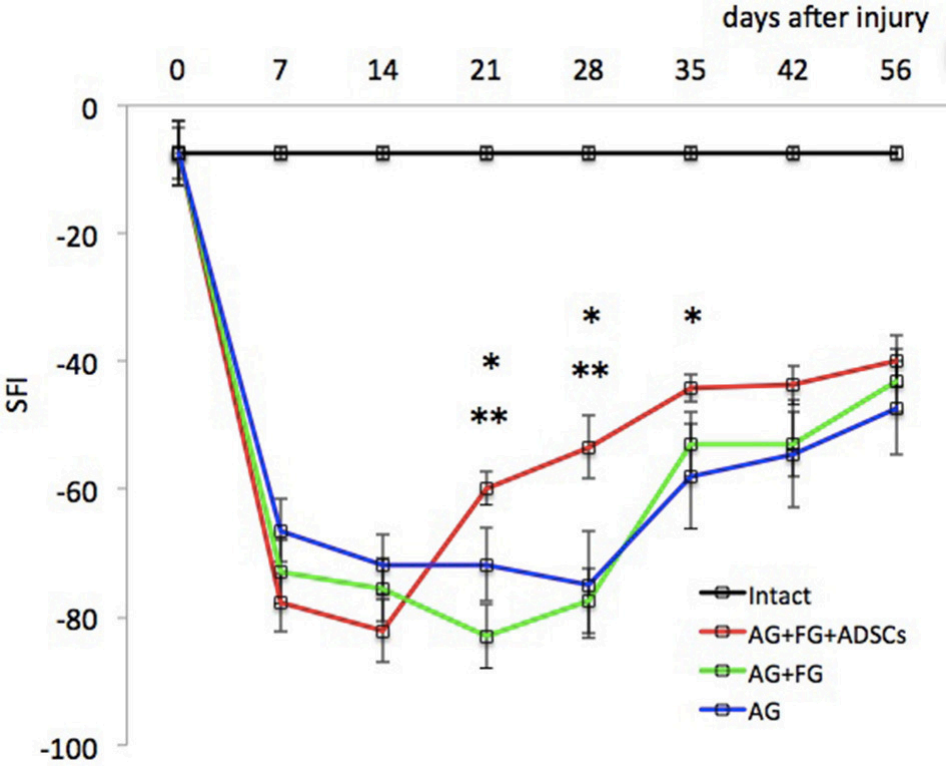
Evaluation of motor function restoration of the hind extremity of rats (Sciatic Functional Index—test). Error bars represent standard error mean. Differences were statistically significant between the groups (*), between groups AG+FG+ADSCs and AG; (**), between groups AG+FG+ADSCs and AG+FG (*, **-*P* < 0.05, one-way ANOVA, Tukey's test).

ADSCs applied together with FG on the area of the sciatic autologous nerve graft stimulated the recovery of extremity motor function. At the late stages, the parameters of motor activity in the AG+FG+ADSCs group increased on average by 26% (*p* < 0.05) as compared to the AG group and by 28% (*p* < 0.05) as compared to AG+FG group (Figure 4).

**Figure 5:**
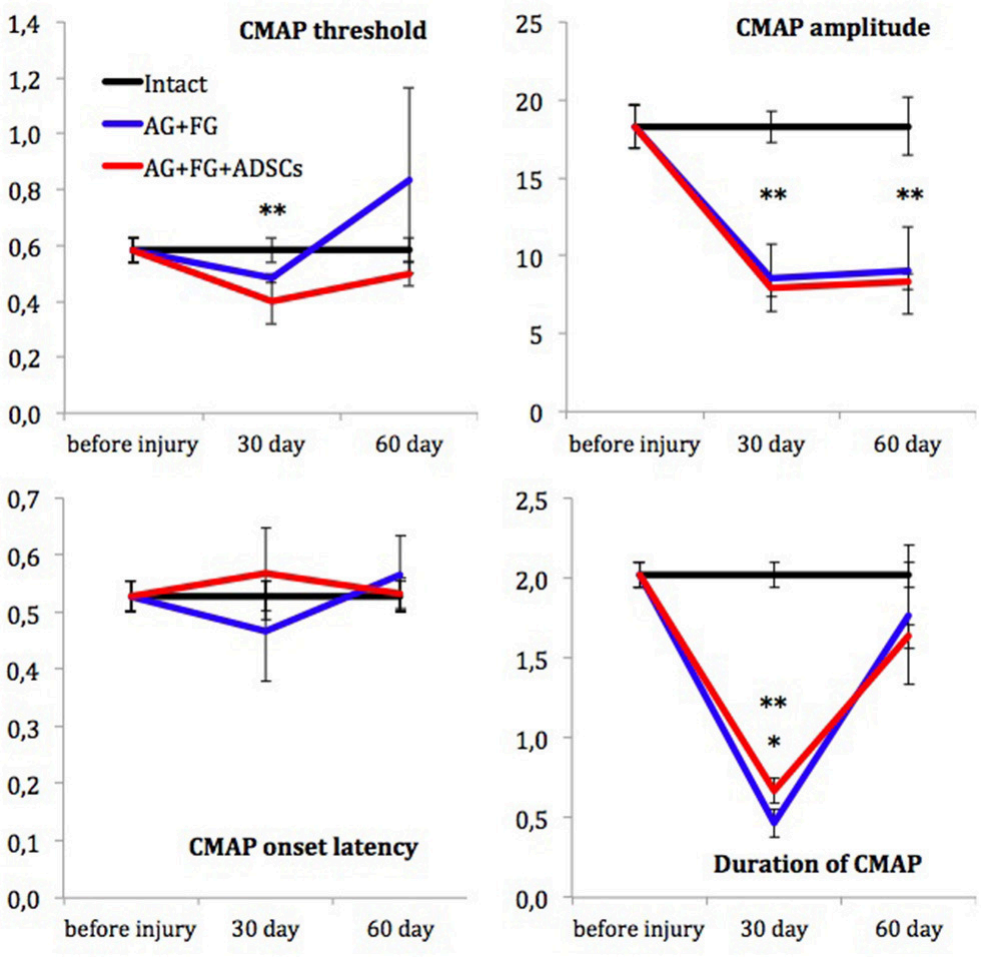
Electroneuromyography measurements. X-axis, testing time: before injury; 30 day and 60 day after injury. Y-axis, CMAP threshold (in volts); CMAP amplitude (in millivolts); CMAP onset latency (in milliseconds) and Duration of CMAP (in milliseconds). Differences were statistically significant between the groups (*), between AG+FG+ADSCs and AG-groups; (**), between experimental and intact groups (*, **-*P* < 0.05, one-way ANOVA, Tukey's test).

Assessment of muscle bioelectric activity did not reveal significant differences in CMAP threshold and CMAP latency among the compared groups on the 30th and 60th days after trauma. Initial CMAP amplitude didn't differ between the AG+FG+ADSCs group, the AG+FG-group, and control group. CMAP amplitude was only 50% from initial on the 30th day after trauma, showing axonal loss in sciatic nerve, persisting up to 60th day in both groups. The duration of CMAP in the groups with sciatic nerve injury decreased substantially 30 days after the injury as compared to the measurements in intact animals but at the same time the CMAP duration was significantly higher in the AG+FG+ADSCs group than in the AG+FG-group. Unlike the CMAP amplitude, the CMAP duration increased and reached the control group level in the 60 days after trauma. No differences in CMAP duration revealed between the AG+FG+ADSCs group and the AG+FG-group (Figure 5).

**Figure 6:**
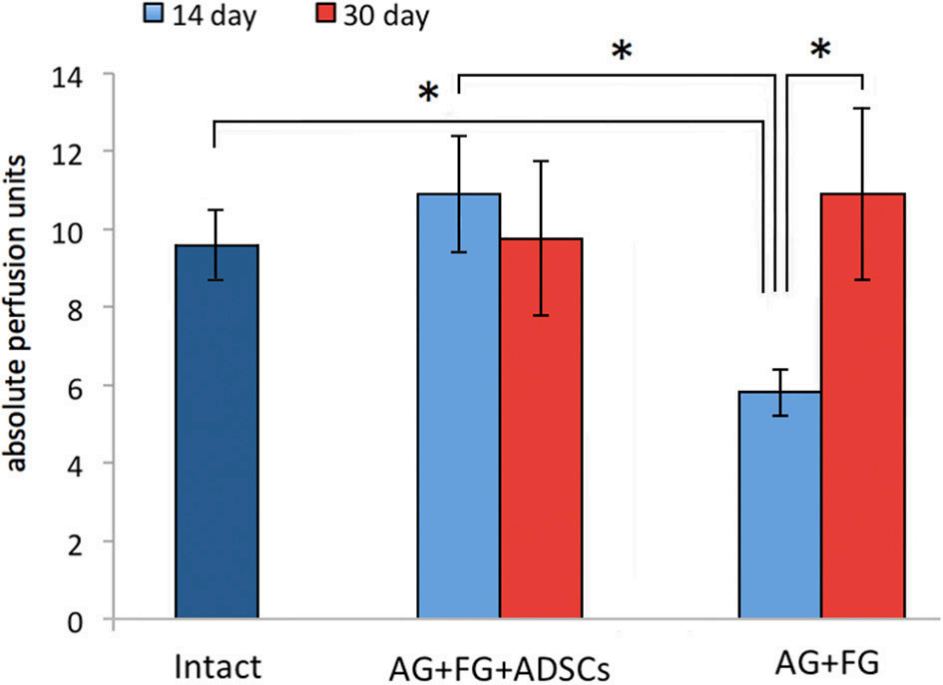
Indicators of vascularization of the distal stump of the sciatic nerve at 14 and 30 days after AG. Differences were statistically significant between the groups *, between experimental groups (*P < 0.05, one-way ANOVA, Tukey's test).

When assessing the restoration of blood supply by laser doppler, the vascularization parameters in the AG+FG+ADSCs group were already restored at 14 days after the injury to levels similar to those of intact animals, whereas in the AG+FG group the blood supply of the distal nerve segment was still decreased. However, no difference among the groups was found after 30 days (Figure 6).

**Figure 7:**
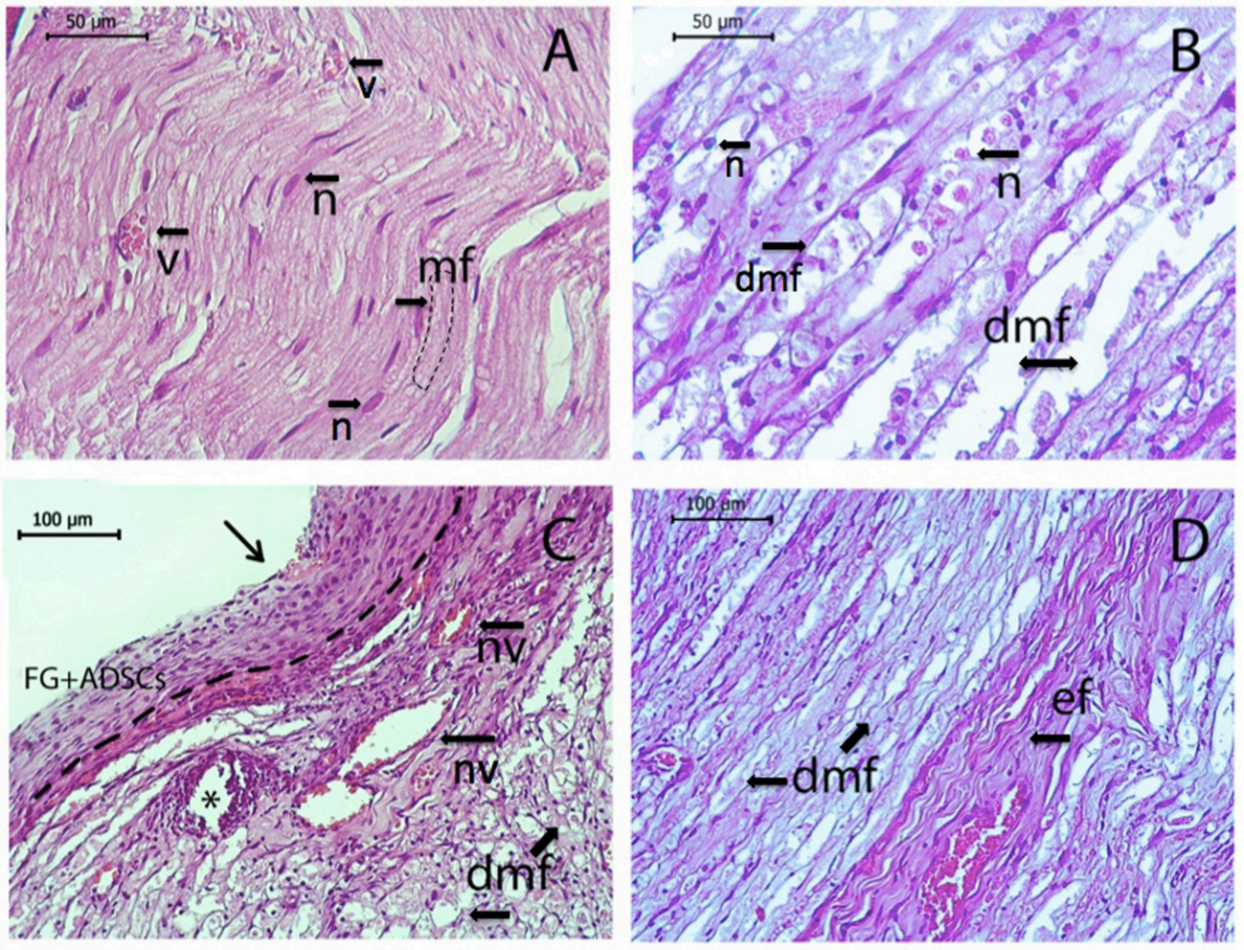
Longitudinal sections of rat sciatic nerve. Intact nerve (*n*, nuclei of Schwann's cells; v, blood vessel; mf, normal myelin fibers) is shown in **(A)**, sciatic nerve 7 days after the operation is shown in **(B–D)**. **(B)** Autotlogus nerve graft area showing degenerated myelin fibers with oedema (dmv) with degenerated nuclei of Schwann's cells (*n*). **(C)** Location of nerve anastomosis—central part indicated by arrow, *the needle hole, a dotted line separates epineurium from the top-located fibrin glue with ADSCs, macrophage aggregation, necrotic altered blood vessels (nv) with a hemorrhage area, and degenerated myelin fibers (dmf) under the epineurium. **(D)** Distal segment of the nerve with degenerative changes of myelin fibers (dmf) and elastic fibers (ef). Hematoxylin and eosin staining. Scale bar **(A,B)** 50 μm, **(C,D)** 100 μm.

Morphological examination of the sciatic nerve longitudinal sections revealed several changes associated with the injury when compared to the intact nerve (Figure 7A). Myelin fibers were found degenerated, with myelin breakdown in the area of the autologous nerve grafting and areas close to graft oedema. Schwann cell nuclei acquired a damaged round shape and were displaced to the periphery of the cylinder (Figure 7B). After transplantation, analysis of the anastomosis area revealed migration of macrophages under the epineurium and areas of hemorrhage with necrotic changes of small vessels (Figure 7C). Importantly, the epineurium preserved its integrity and residues of fibrin glue with large-sized cells and clearly visible nuclei on its surface were found in the AG+FG+ADSCs group (Figure 7C). Degenerative changes spread both to central and distal directions. Here, destructive changes were observed not only in myelin fibers, but also in elastic fiber that acquired a denser and more convoluted structure (Figure 7D). The transplantation of ADSCs with fibrin glue significantly improved the described degenerative changes.

**Figure 8:**
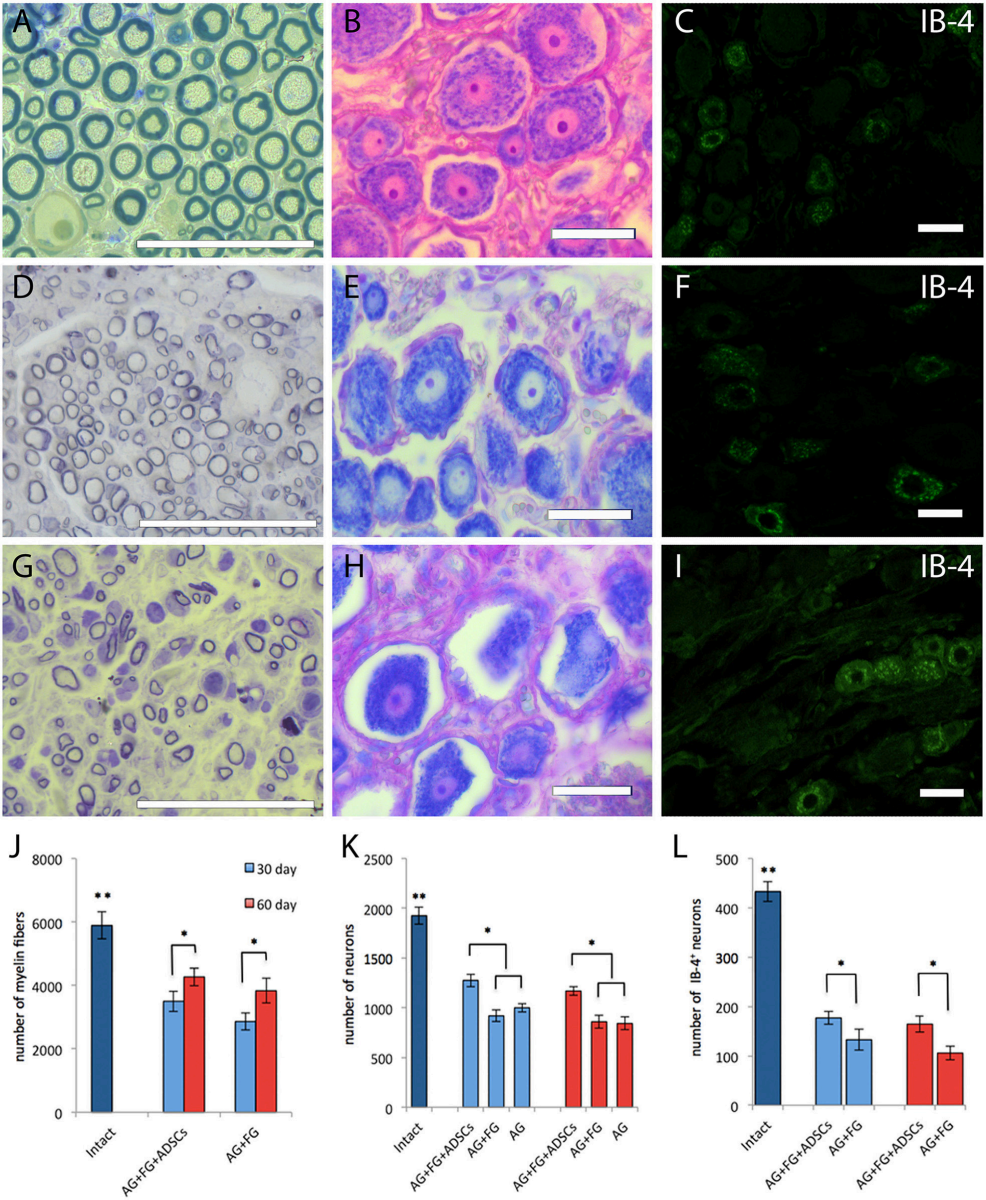
Morphology and number of fibers and neurons in intact and transplanted animals. Top line **(A–C)**, intact animals; second line **(D–F)**, AG + FG + ADSCs group 30 days after the injury; third line **(G–I)**, AG + FG group 30 days after the injury. Scale bar scale 50 μm. **(A,D,G)** Light microscopy images of myelinated fibers fixed in osmium and stained by methylene blue. **(B,E,H)** Light microscopy images of L5 DRG neurons stained by azure-eosin. **(C,F,I)** Confocal microscopy images of IB-4+ neurons stained with the fluorescent cytoplasmic marker Isolectin-B4 [lectin from Bandeiraea simplicifolia (BSi-B4)]. **(J)** Number of myelin fibers in the distal stump of the sciatic nerve; **(K)** Number of L5 DRG neurons; **(L)** Number of IB-4+ neurons L5 DRG. Differences were statistically significant between the groups (*) between experimental groups, (**) between intact and experimental groups (*, **-*P* < 0.05, one-way ANOVA, Tukey's test).

Myelin fibers in the distal nerve segment 30 days after the autologous nerve grafting were represented by round masses with the much smaller diameter than that of intact animals (Figures 8A,D,G). However, the number of myelin fibers in the nerve distal segments 30 days after the surgery in the AG+FG+ADSCs group was increased by around 18 % (*p* < 0.05) compared to the AG+FG group. The number of myelin fibers in the both groups was increased by a further 20% 60 days after the surgery compared to day 30 (*p* < 0.05) (Figure 8J). The number of neurons of the L5 spinal ganglion decreases in all groups after the injury as compared to intact animals. The number of neurons decreased even further by 60 days after the surgery. Although chromatolysis was visible in the neurons 30 days after the sciatic nerve autografting (Figures 8B,E,H), the number of neurons of the L5 spinal ganglion was significantly higher in the AG+FG+ADSCs groups compared to AG+FG and AG. The same increase was found 60 days after the surgery, demonstrating the regenerative capacity of the grafted ADSCs (Figure 8K).

Assessment of the IB4+ neurons after transplantation demonstrated that IB4 expression was found not only in small (up to 30 μm in diameter) but also in medium-sized (up to 50 μm) neurons (Figures 8C,F,I). Although, an expected decrease in the number of IB4+ positive cells was found in injured animals, a significant increase of IB4+ neuron was found in the AG+FG+ADSCs group both at day 30 and day 60 after transplantation compared to the AG+FG control group (Figure 8L).

Therefore, altogether the changes observed after autologous transplantation demonstrated the enhanced regeneration capacity of ADSCs in fibrin glue that resulted in motor function recovery after injury.

## Discussion

Autologous nerve grafting is the gold standard procedure to ensure nerve continuity restoration in cases of diastasis after experimental injury induced to model neurodegenerative and regenerative post-traumatic processes. Indeed, the autologous conduit is biocompatibe and contains autologous Schwann cells and extracellular matrix structural proteins contributing to neuron survival and stimulating the regeneration of damaged axons (36, 37). Using functional tests, electroneurophysiology assessment, vascularization assessment, and morphometric study of the sciatic nerve and L5 spinal ganglion, in this study we were able to confirm that surgery aimed at approximating the ends of an injured nerve is not enough to achieve complete regeneration. Therefore, regeneration stimulation by means of application of autologous ADSCs to the area of nerve injury allowed a significant enhancement of regeneration through stimulation of angiogenesis and neuroprotection.

The rate and degree of nerve fiber de- and regeneration after damage is a complex process associated with inflammation, adhesion, regulation of neurotrophic factors, neurotransmitter synthesis and release, formation of the nerve growth cone, neuron survival, growth of axons and myelination, and many other factors (38, 39). At the same time, immune reactions taking place in the injury area cause the expression of pro-inflammatory transmitters around the damaged tissue and contribute to the development of scar tissue, nerve fibrosis (40), neuromas, and hyperplasia which impact on nerve damage and on conduction restoration after injury (41).

During nerve fiber regeneration the central segment of the neuronal axon interacts with glial components and grows according to the concentration gradient of chemical factors against the background of reactive changes in the sensory neurons and degenerative processes in the peripheral segment of the nerve. The success of reinnervation depends on the ability of axons to reach their target organ. It is known that the sciatic nerve is comprised of processes of motor and sensory neurons with trophic function. If there is no contact with the axon, Schwann cells activate the synthesis of neurotrophic factors such as nerve growth factor (NGF), fibroblast growth factor (bFGF), and the ciliary neurotrophic factor (CNTF) (42, 43). These factors, together with ATP and neuregulin released from the proximal nerve ends (44, 45) promote the formation of new Schwann cells (46) and, together with acetylcholine, stimulate their further proliferation (47).

Functional stimulation of the innervated area stops when the sciatic nerve is transected, and the secretion or action of co-transmitters and trophogens is increased (48). From the survival of neurons directly depends on the speed and quality of recovery of the motor and sensitive function of the injured extremity. We demonstrate that on days 7 and 14, the recovery of motor function in the AG+FG+ADSCs and AG+FG groups is lower than in the AG group, however, it is very early to talk about any therapeutic effect on these periods. This fact at this time is due to the mechanical effect of FG on the sciatic nerve. Normally, the sciatic nerve passes freely between the muscles, and the placement of fibrin glue on its surface leads to its adhesion to the muscles, creating the effect of scarring. The result of the influence of the ADSCs observed in 14 days after the injury, when the cells that have penetrated into the nerve thickness begin to show their therapeutic effect. Neuron regenerative potential is preserved 2 to 15 days after nerve transection and up to several months after nerve crush. This interval is believed to be the most favorable for regeneration stimulation and support of neuron survival (49, 50).

Absolute difference in CMAP duration between AG+FG+ADSCs and AG+FG groups is not big despite statistical significance that rise the question of biological meaning of this observation. But they correspond with higher results of motor activity in AG+FG+ADSCs group from 21 to 35 days after trauma. Therefore, we suppose that this difference reflects higher amount of motor axons of sciatic nerve in AG+FG+ADSCs group.

Therefore, the assessment of the number of spinal ganglion neurons after the injury is essential for understanding the mechanisms of spontaneous sciatic nerve regeneration, estimation of quality of surgical procedures aimed at nerve structure restoration, and methods of stimulation of post-traumatic regeneration. L4-L5 spinal ganglia contain 98–99% of nerve cell bodies whose axons form the rat sciatic nerve (51). It is known that sciatic nerve axotomy results in the death of spinal ganglion neurons (52–55) and we demonstrated low neuron survival rate in the L5 spinal ganglion up to day 60 after the injury. When assessing the total neuron count, it was found that small diameter pain neurons are more vulnerable.

We showed that after sciatic nerve injury IB4 expression is found not only in small pain neurons but also in medium-sized neurons. IB4 is believed to be a marker of small nociceptive neurons that have unmyelinated processes in the sciatic nerve innervating the epidermis and showing lower survival both *in vitro* and *in vivo* (56). Therefore, this type of neurons is more prone to cell death as compared to medium and large diameter neurons, which is consistent with our results. Sensory IB-4+ neurons are less flexible and grow worse after axotomy. After sciatic nerve injury, IB4 neuron terminal axons can show retraction (57) and have substantially lower regeneration ability (58). It is therefore likely that IB4 expression in some medium diameter neurons after nerve injury could be due to axon growth inhibition.

Although molecular mechanisms underlying the death of spinal ganglion neurons in response to nerve axotomy are not fully understood, there is enough evidence of the role played by injury severity on neuron apoptosis and potential ability of axons to grow to respective distal receptors (59). Exogenous trophic factors can counteract the post-traumatic death of sensory neurons. Transplanted ADSCs may produce neurotrophic factors as cells transplanted into the nerve show retrograde neuroprotective effect on respective sensory neurons (52, 53). The beneficial effect of cell therapy can be seen in as little as the first week after the surgery (60). Therefore, it can be speculated that the presence in an injured nerve of exogenous progenitor cells with high paracrine activity in the first several days after the injury promotes axon growth and survival. Indeed, we did observe a significant increase in the number of myelin fibers L5 and IB4+ neurons after ADSCs transplantation, suggesting a trophic action of MSCs after injury.

In addition, morphological assessment of nerve structure at early stages after the injury revealed inflammation both in groups with cell stimulation and in control groups. The severity of inflammation was, however, much lower in the group treated with ADSCs.

In order to assess the extent of regeneration and its clinical relevance, we performed functional tests as the examination of morphological changes in the injured sciatic nerve often showed discrepancy between histopathological changes and functional observations (61).

We showed that nerve reconstruction by means of autologous nerve repair considerably reduces the ability of the operated extremity to move. It may be that nerve injuries go hand in hand with neuropathic pain (62) but we demonstrated that ADSCs can induce a significant increase in the sciatic functional index and associated motor function recovery.

Several studies demonstrated that transplanted MSCs can directly influence angiogenesis by influencing all the stages of vessel formation and maturation (63). Indeed, MSCs secrete cytokines and growth factors stimulating survival, growth, and differentiation of vascular endothelium (64), thus preventing the formation of neurotrophic ulcers caused by vessel destruction and ischemia. It is likely that the restoration of blood flow in the operated sciatic nerve could be associated with the activation of proangiogenic factor expression by transplanted ADSCs, such as fibroblast growth factor and endothelial growth factor (65, 66).

With the help of nerve conduction studies, we were able to assess the functioning of sciatic nerve motor fibers. Since fast-conducting fibers, such as axons of motor neurons (type Aα) and afferent muscle fibers (type Aβ), are thicker than slow-conducting, the process of their recovery takes more time. Unmyelinated neuron axons (type C), such as axons of the spinal ganglion sensory neurons, involved in the transmission of pain (67), temperature, and postganglionic sympathethic transmission are the slowest ones (68). Decreased amplitude and shortened CMAP duration by day 30 reflected the loss of sciatic nerve axons, most probably due to wallerian degeneration. In the period from day 30 to day 60 no increase in the CMAP amplitude was registered but the CMAP duration increased, thus demonstrating the increasing number of functionally active axons in the experimental and control groups. These changes also evidenced incomplete remyelination of regenerated axons and subsequent CMAP dispersion, which at early stages of re-innervation caused increased CMAP duration with unchanged amplitude. Significantly longer CMAP duration in the experimental group as compared to the control group by day 30 could be a sign of earlier regeneration under the influence of ADSCs, which is in line with the data we obtained for the number of myelin fibers in the distal nerve segment. The number of myelin fibers in the distal sciatic nerve segment increased in all studied groups but this parameter was higher in the group with ADSCs.

Altogether, our morphological and functional data demonstrate the beneficial effect of cell therapy with ADSCs. ADSCs transplanted with FG which facilitate the joining of the segments of the transected sciatic nerve, entered under the epineurium through the junctions of nerve segments, directionally migrated predominantly retrogradely, contributed to the sensory neurons survival through stimulating the growth of their axons, and promoted conduit vascularization by restoring the motor function of the injured extremity.

We cannot rule out a possible transdifferentiation of ADSCs into Schwann cells which could be responsible for the augmented regeneration observed; this possibility, however, warrants further investigation. Taking into account that Schwann cells play a key role in peripheral nerve survival and functioning (68), we believe that transplantation of ADSCs provides the optimal conditions for regeneration. Importantly, cell transplantation should be carried out in the acute traumatic period with minimally invasive delivery by application of potential regeneration stimulants as part of fibrin glue in order to achieve effective repair of peripheral nerve damage.

## Conclusion

Our data suggest that MSCs transplanted in fibrin glue to the place of nerve injury have a neuroprotective effect on DRG L5 sensory neurons, stimulate axon growth, and myelination. We propose a new method of MSCs delivery to the area of traumatic injury by using fibrin glue. This method of delivery of regeneration stimulants could be beneficial for the successful treatment peripheral nerves injuries and easily translate to the clinical practice.

## Ethics Statement

This study was carried out in accordance with the recommendations by the Local Ethics Committee of Kazan Federal University. The protocol was approved by the Local Ethics Committee of Kazan Federal University (Permit Number 5 of May 27, 2014).

## Author Contributions

RM: experimental design, collection and assembly of data, data analysis and interpretation, manuscript writing, and final approval of manuscript; GM: assembly of data, histology, data analysis and interpretation, manuscript writing; AM: work with animals, histology, functional tests, sample preparation; MZ: working with cell culture, data collection, manuscript writing, manuscript editing; AS: nerve conduction studies; ARo: nerve conduction studies, data analysis, and interpretation; VS: work with animals, acquisition of data, data processing; DA: vascularization assessment, data analysis, manuscript editing; AZ: assisted with data collection, manuscript editing; KI: work with animals, data collection; CA: conception and design, manuscript editing, and final approval of manuscript; AK: manuscript editing, and final approval of manuscript; ARi: financial support, manuscript editing, and final approval of manuscript. All authors provided critical feedback and helped shape the research, analysis and manuscript.

### Conflict of Interest Statement

The authors declare that the research was conducted in the absence of any commercial or financial relationships that could be construed as a potential conflict of interest.

## Supplementary Material

Supplementary Figure 1 Schematic of the rat walking the track analysis; TOF (e), distance from the experimental foot toes to the intact foot toes; TOF (*n*), distance from the intact foot toes to the experimental foot toes; PL, length of the footprint from the heel to the third toe of the same foot; TS, distance between the first and the fifth toes of the same foot; IT, distance between the second and the fourth toes of the same foot, e, experimental; n, normal.
