# Supplementary figures and images for: Adipose-Derived Mesenchymal Stem Cells Applied in Fibrin Glue Stimulate Peripheral Nerve Regeneration
Source: Front Med (Lausanne). 2019 Apr 9;6:68. doi: 10.3389/fmed.2019.00068 (PMC6465797; doi:10.3389/fmed.2019.00068)

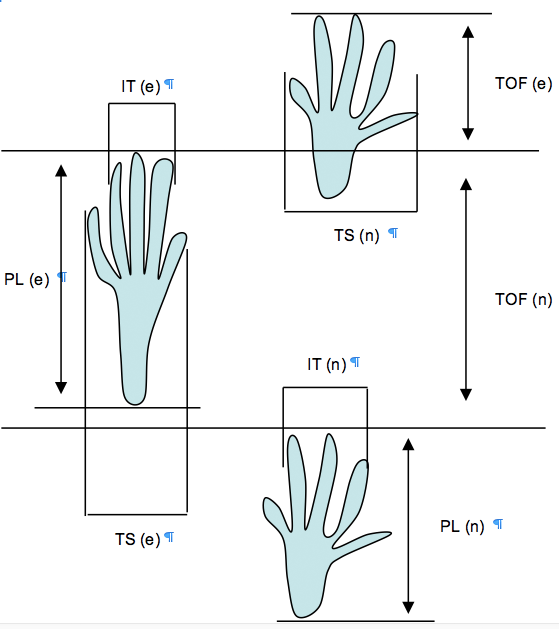

Supplement: Supplementary Figure 1 — Schematic of the rat walking the track analysis; TOF (e), distance from the experimental foot toes to the intact foot toes; TOF (n), distance from the intact foot toes to the experimental foot toes; PL, length of the footprint from the heel to the third toe of the same foot; TS, distance between the first and the fifth toes of the same foot; IT, distance between the second and the fourth toes of the same foot, e, experimental; n, normal. [file Image_1.tif]
